# Serum free testosterone level in coronary artery disease in candidates for coronary artery bypass graft surgery: A cross-sectional study

**DOI:** 10.18502/ijrm.v19i3.8577

**Published:** 2021-03-21

**Authors:** Shahriar Mali, Kurosh Irani, Seyed Mohammad Mohammadi, Mohammadtaghi Sarebanhassanabadi

**Affiliations:** ^1^Yazd Cardiovascular Research Center, Shahid Sadoughi University of Medical Sciences, Yazd, Iran.; ^2^School of Medicine, Shahid Sadoughi University of Medical Sciences, Yazd, Iran.

**Keywords:** Coronary artery disease, Testosterone, Coronary artery bypass graft surgery.

## Abstract

**Background:**

Due to the controversy over the effect of serum testosterone levels on coronary artery diseases, this survey explores the serum levels of free testosterone, luteinizing hormone, and follicle-stimulating hormone in candidates for coronary artery bypass graft compared with an age-matched control group and evaluates the associated factors in these participants.

**Objective:**

To determine the testosterone level in elective coronary artery bypass grafting participants.

**Materials and Methods:**

In this cross-sectional study, all male patients aged > 40 yr as candidates for elective coronary artery bypass grafting, who were referred to the Afshar Hospital, Yazd, Iran, from March 2018 to March 2019, were included. In total, 100 men were enrolled (50 cases and 50 controls). Their serum levels of free and total testosterone, luteinizing hormone, and follicle-stimulating hormone were measured and the results were compared.

**Results:**

The findings indicated a significant difference between the two groups in total and free testosterone (both p < 0.001); they were lower in the case group. There was also a significant difference in the total testosterone of the participants with diabetes mellitus compared with no-diabetic individuals (p = 0.007). Free testosterone of diabetic subjects taking insulin was lower compared with those taking no insulin (p = 0.04). There was also an association between the body mass index and free testosterone, left ventricular ejection fraction and total testosterone, and a significant and negative relation between the duration of hospital admissions and free testosterone (p < 0.05).

**Conclusion:**

This study illustrates that participants with coronary artery disease bear a significantly low testosterone level in comparison with the healthy control group.

## 1. Introduction 

Over the past decades, the pattern of the most common fatal diseases in adults has changed from communicable to non-communicable diseases, of which cardiovascular diseases (CVDs) appear to be highly important with regard to global mortality as well as disease burden (1). As estimated, in 2016, about 40.5 million worldwide deaths (71%) emerged from non-communicable diseases, 80% of which emanated from cancers, CVDs, chronic respiratory diseases, and diabetes mellitus (DM) (2); however, interestingly, about half of these cases have arisen from CVDs alone (3). Although several strategies have been suggested to shrink the mortality rate of CVDs resulting in 39% decrease in the age-related mortality, the global deaths from CVDs have increased by 41% over the past two decades (from 1990 to 2013), primarily as a result of population growth (resulting in 55% increase in the mortality rate) and population aging (resulting in 25% increase in the mortality rate) (4). Accordingly, research has been advanced toward the risk factors of CVDs, specifically coronary artery diseases (CADs) as the main cause of mortality of CVDs in order to develop the preventive strategies for CVDs (5, 6).

Moreover, studies on the risk factors of myocardial infarction (MI) and CVDs have touched upon the participants' sex as a critical and determinant indicator (7). It has been identified that women experience their first MI about 10 years later than men, while this difference appears to attenuate after menopause (8). A body of evidence also suggests that this difference projects likely as the result of altered immune response during atherosclerosis triggered by sex hormones (9). Androgenic hormones may play a significant role in the onset and progression of CVD in men as testosterone produces vasorelaxation, improves the endothelial function, and has an atheroprotective effect resulting in a lower risk of MI in older men after andropause (10, 11). However, results of a meta-analysis suggest the controversy on the effect of testosterone-replacement therapy on older men around the incidence of MI (12).

On the basis of these controversial results, the study of the association of serum levels of free T with CADs appears to be necessary. In Iran, the incidence rate of MI in men seems to be three times higher than in women but the effect of sex hormones on the risk of MI remains yet to be known (13).

This study, therefore, deals with a survey concerning the serum levels of free testosterone, luteinizing hormone (LH), and follicle-stimulating hormone (FSH) in participants who were candidates for coronary artery bypass graft (CABG) compared with an age-matched control group.

## 2. Materials and Methods

### Study design

In this cross-sectional study, all male participants who were candidates for elective CABG and were referred to the Afshar-Yazd Hospital, Yazd, Iran, between March 2018 and March 2019 were considered as the study population. The sample size was calculated as 50, considering 95% confidence interval (CI), ± 1 SD for serum-free testosterone, and 1.1 unit error. The participants meeting the inclusion criteria of the study were included using the consecutive method. The inclusion criteria comprised of male participants aged > 40 yr; candidates for elective CABG during the study period. Those who did not have any positive history of MI; emergency CABG, testicular surgery or tumor, chemotherapy, hepatorenal failure; and did not show any history of taking exogenous testosterone or any other hormonal drugs such as finasteride and flutamide were excluded. Also, we had a control group including 50 participants with no history of coronary artery diseases.

Personal particulars of the participants were recorded through a checklist designed for data collection including participants' demographics such as age, body mass index (BMI), history of diseases including DM, hypertension (HTN), hyperlipidemia (HLP), smoking, drug abuse, as well as taking aspirin, beta-blockers, statins, and insulin. Before surgery, a venous blood sample was taken from all participants after 12 hr of fasting in a sitting position. The blood samples were obtained and transferred to the hospital laboratory immediately. The blood samples were centrifuged at 3000 rpm for 5 min at room temperature using a SELECT LAB (Model: TD25-WS, Lu Xiangyi, China). The serum levels of free and total testosterone, LH, and FSH were measured in the lab using ELISA test. To measure FSH and LH, LIASON instrument (Type: 2229, Liaison Diasorin, Germany) was used. The normal range of the kits was 2.8-6.8 mIU/mL for LH and 1.3-11.8 mIU/mL for FSH. The total and free testosterone were measured by MINDRAY microplate ELISA reader (Model: MR-96A, Mindray, Germany). The normal range of the kits was 2-11 ng/mL for total testosterone and 5.7-30.7 pg/mL for free testosterone.

Of note, all participants underwent echocardiography and angiography; however, we did not record the severity of coronary artery involvement as we did not have access to films. After the surgery, hemodynamic instability (BP < 60 mmHg, the need to adrenalin > 0.1 µg/kg/min, or noradrenalin > 0.01 µg/kg/min, and/or dobutamine > 8 µg/kg/min), as well as mechanical support (intra-aortic balloon pump) and cases of death during hospital admissions were extracted from the participants' medical records.

The results were finally compared with the control group comprising of 50 participants with no history of coronary artery diseases referred to the same laboratory.

### Ethical considerations

This study was approved by the ethics committee of the Shahid Sadoughi University of Medical Sciences in Yazd, Iran (IR.SSU.MEDICINE.REC.1395.252). All participants were informed about the study design and objectives and had signed a written informed consent form prior to the study.

### Statistical analysis

The results of the numerical variables are presented as mean ± standard deviation (SD) and compared between the groups using *t* test or Mann-Whitney U test whenever the data failed to show normal distribution or when the assumption of the equal variances was violated across the study groups. Categorical variables were reported by a number (percentage) and compared between the groups using Chi-square test. For comparison, the total T levels of the case group were categorized into three groups: < 2 ng/mL (lower limit normal: group A), 2-7.5 ng/mL (group B) and 7.6-11 ng/mL (group C) while similarly free testosterone was classified into three levels according to their values: < 5.7, 5.7-18.2, and 18.3-30.7 ng/mL as groups A-C. The association of variables was tested by Pearson's or Spearman's correlation coefficient. For statistical analysis, IBM SPSS for Windows version 22.0 (IBM Corp. 2013. Armonk, NY: IBM Corp.) was used. P-values < 0.05 were considered as statistically significant.

## 3. Results

Of the 100 participants included in this study, 50 were evaluated as the case and 50 as the controls. The mean ± SD of the participants' age in the case and control groups turned out to be 61.5 ± 8.48 years (range: 42-79 yr) and 61.72 ± 8.37 yr, respectively; no significant difference was thus identified in this regard (p = 0.896). Moreover, no significant difference could be found between the mean ± SD of the participants' BMI in the groups (28.46 ± 19.5 vs 25.61 ± 3.95 kg/m${}^{2}$; p = 0.313).

Of the 50 participants in the case group, 45 underwent off-pump CABG and five were treated with on-pump CABG. The mean ± SD of the participants' left ventricular ejection fraction (LVEF) in the case group was 43.90 ± 8.09 (25-60%). The frequency of positive underlying diseases in the case group was as follows: 46% had DM, 34% HTN, 36% HLP, 26% were currently smoking, and 26% had drug abuse. Considering the medical history, 54% had a positive history of using beta-blockers, 66% statins, and 8% antidiabetes drugs. After the surgery and in the intensive care unit (ICU), 4% required a balloon pump and 30% were in need of inotropes with no fatality. The mean ± SD of hospitalized participants in the case group was 5.6 ± 1.3 (range: 4-11) days, that in the ICU was 3.6 ± 0.9 (range: 2-7) days, and that of the intubation period proved to be 5.01 ± 0.60 (range: 3-21) hr.

Comparing the mean values of free and total testosterone between the participants with and without any underlying disease and medical history of drug-taking using *t* test illustrated a significant difference in the total testosterone of the participants with DM (p = 0.007) and in the free testosterone of the patients with a history of taking insulin (p = 0.04) (Table I). Studying the correlation between the variables revealed a significant positive relationship between the BMI and free testosterone (p = 0.023), LVEF and total testosterone (p = 0.02), and a significant but negative association between hospitalization and free testosterone (p = 0.026) (Table II).

As set out in Table III, comparing the serum parameters between the case and control groups using *t* test indicated no difference in the mean values of LH (p = 0.418) and FSH (p = 0.519), while a significant difference was identified between the groups in terms of mean values of total and free testosterone (p = 0.001).

Categorizing participants' total testosterone into three groups revealed 13 participants belonging to group A level of total testosterone, 36 to group B, and only one patient to group C. Also categorizing participants' free T into three groups in the cases revealed 20 participants being in group A level of free T, 30 others in group B, while no participant was found to be in group C.

**Table 1:** Comparison of the mean values of free and total testosterone according to the participants' medical history in the case group


**Variables**		**Number**	**Total testosterone (ng/mL)**	**P-value**	**Free testosterone (ng/mL)**	**P-value**
**Total diabetes mellitus**	Positive	23	0.007	2.37 ± 1.34	6.88 ± 3.43	0.36
	Negative	27		3.60 ± 1.66	7.77 ± 3.47	
**Hypertension**	Positive	13	2.81 ± 1.35	0.48	6.99 ± 3.01	0.59
	Negative	33	3.15 ± 1.76		7.55 ± 3.68	
**Hyperlipidemia**	Positive	14	3.44 ± 1.44	0.27	7.58 ± 3.28	0.78
	Negative	36	2.87 ± 1.68		7.28 ± 3.55	
**Smoking**	Positive	13	3.00 ± 2.45	0.937	7.14 ± 4.37	0.79
	Negative	37	3.04 ± 1.26		7.44 ± 3.13	
**Substance abuse**	Positive	13	2.63 ± 1.17	0.37	6.65 ± 3.92	0.41
	Negative	37	3.11 ± 1.74		7.61 ± 3.35	
**Aspirin**	Positive	17	3.36 ± 1.69	0.05	6.59 ± 3.17	0.26
	Negative	33	2.41 ± 1.33		7.76 ± 3.56	
**Beta-blockers**	Positive	27	2.86 ± 1.42	0.41	7.39 ± 3.19	0.94
	Negative	23	3.24 ± 1.85		7.33 ± 3.80	
**Statins**	Positive	33	3.05 ± 1.42	0.91	7.35 ± 3.21	0.97
	Negative	17	3.00 ± 2.02		7.38 ± 3.98	
**Insulin**	Positive	4	1.82 ± 0.43	0.12	4.11 ± 1.56	0.04
	Negative	46	3.14 ± 1.65		7.64 ± 3.43	
**Inotrope**	Positive	15	2.97 ± 1.36	0.86	6.96 ± 3.45	0.59
	Negative	35	3.06 ± 1.75		7.53 ± 3.48	
**Intra-aortic pump**	Positive	48	2.71 ± 0.73	0.77	6.04 ± 1.92	0.58
	Negative	2	3.05 ± 1.66		7.42 ± 3.50	
Data presented as Mean ± SD. To compare variables, *t* test was used						

**Table 2:** The results of Pearson's correlation test between the variables of the case group


	**Age**	**BMI**	**Total testosterone**	**Free testosterone**	**LVEF**	**Duration of hospital admissions**	**Admission in ICU**
**BMI**	r = 095 p = 0.511	–	–	–	–	–	–
**Total testosterone**	r = –0.089 p = 0.540	r = –0.002 p = 0.989	–	–	–	–	–
**Free testosterone**	r = –0.247 p = 0.084	r = 0.321 p = 0.023	r = 0.314 p = 0.026	–	–	–	–
**LVEF**	r = 0.105 p = 0.470	r = 0.076 p = 0.601	r = 0.328 p = 0.020	r = 0.044 p = 0.761	–	–	–
**Duration of hospital admissions**	r = 0.212 p = 0.139	r = –0.215 p = 0.133	r = 0.063 p = 0.666	r = –0.314 P = 0.026	r = 0.025 p = 0.861	–	–
**Admission in ICU**	r = 0.107 p = 0.461	r = –0.127 p = 0.380	r = 0.071 p = 0.623	r = –0.224 p = 0.119	r = 0.035 p = 0.812	r = 0.576 p < 0.001	–
**Duration of intubation**	r = 0.082 p = 0.576	r = 0.099 p = 0.501	r = 0.006 p = 0.969	r = –0.079 p = 0.588	r = –0.099 p = 0.500	r = 0.195 p = 0.179	r = 0.310 p = 0.030
Values show the Pearson's correlation coefficient and p-values were considered significant when < 0.05 BMI: Body mass index, LVEF: Left ventricular ejection fraction, ICU: Intensive care unit							

**Table 3:** Comparison of the results of serum parameters between the case and control group


[36.96mm,lr]**Variables** **Groups **	<statement> <title>Case </title> </statement>	**Control**	**P-value**
**LH**	6.38 ± 5.97	7.01 ± 6.34	0.418
FSH*	11.34 ± 13.33	10.75 ± 9.13	0.519
**Total testosterone (ng/mL)**	3.03 ± 1.63	4.16 ± 1.31	0.001
**Free testosterone (ng/mL)**	7.36 ± 3.45	11.26 ± 4.08	0.001
Data presented as Mean ± SD for continuous variables, *t* test was used to compare the continuous variables, LH: Luteinizing hormone, FSH: Follicle-stimulating hormone *Median (Interquartile Range); Case: 7 (4.39-13.57), Control: 7.88 (5.82-11.52)			

## 4. Discussion 

The results of this study suggested the serum level of free and total testosterone in the group candidate for CABG being significantly lower compared with the control group. We included all participants > 40 yr to reduce the chance of genetic factors inducing CAD which are usually observed at younger ages. Furthermore, we did not include the patients with recent MI or any other major stress so as to eliminate the effect of this factor on the androgen-level change. The results of our study indicated no patient in the case group having upper-limit total or free testosterone or between mid-range and upper-limit free testosterone. These results suggest that participants with CAD bear a lower level of testosterone with regard to their age and BMI matched with the control group, thereby confirming the results reported by other researchers who suggest lower level of serum testosterone in middle-aged men with CAD including the participants with stable or unstable angina pectoris or acute MI (14).

In an Iranian study, Allameh and colleagues evaluated 200 participants in the CAD group and 135 individuals in the control group and reported significantly higher dehydroepiandrosterone sulfate (DHEA-S) and testosterone levels in the control group (15), hence being in line with our results. In their study, the serum testosterone level appeared to be 0.47 ± 0.65 ng/mL in the participants with atherosclerotic coronary arteries (N = 35), 0.04 ± 0.08 ng/mL in those with the history of acute MI (N = 30), and 1.12 ± 0.29 ng/mL in healthy subjects (N = 30) (15).

In another study, Malkin and colleagues suggested that testosterone deficiency is a common finding in participants with CAD, being inversely associated with their survival (16); this is consonant with the results of the present study as we too did not notice any patient with an upper-limit total or free testosterone or between mid-range and upper-limit free testosterone levels. Although a number of aforementioned studies (14-16) have confirmed our findings in terms of lower testosterone levels in the participants with CAD, other studies have reported no difference in the DHEA-S and serum testosterone levels between the case and control groups (17, 18).

In addition to the controversial results regarding the different testosterone values between the participants with CAD and the control group, the association of androgens with the severity of CAD also needs to be tackled. In a study by Hu and colleagues the authors concluded a negative correlation between the serum testosterone and the severity of coronary artery stenosis (14). However, Allameh and colleagues detected no association between the androgenic hormone levels and the risk of CAD (15). In the present study, we did not have access to angiographic assessment results to indicate the association between the serum testosterone and the severity of CAD. Even the results of meta-analysis on this issue have failed to project any definite results (19). However, one meta-analysis concluded the results of studies published before and after 2007 being different. Recent studies suggest that endogenous testosterone is not associated with the risk of CVD in middle-aged men, and instead may poorly protect the elderly against CVD (20). However, we did not separate the results of the elderly although we included the participants aged > 40 yr to reduce the confounding effect of age on the results. To balance, it is well-known that T has vasoactive properties and it is not easy to rule out the low testosterone in the participants with CAD, which suggests the discrepancy in the results attributed to different laboratory measurements, definitions of CAD, as well as the notion that serum testosterone levels may not represent the true serum androgen level (21). Furthermore, several factors may affect the serum testosterone levels that can impact the results (22).

Pieces of evidence suggest the effect of underlying diseases including dyslipidemia, obesity, and DM on the serum testosterone (23, 24). Therefore, in this study we made an attempt to examine the difference in testosterone values based on several diseases such as DM, HTN, and HLP, and the results demonstrated the mean total testosterone levels of the participants with DM and the mean free testosterone of the participants taking insulin being lower than those without. The association of T and DM has also been suggested in previous studies (15, 25), which confirms the results of the present study. Also, lower mean testosterone in the participants who receive insulin confirms the association of diabetes with testosterone and is consistent with the results of the previous studies suggesting the relationship between insulin resistance and low testosterone levels (26-28).

Furthermore, the results of logistic regression in our study exhibited a positive association between BMI and free testosterone, between LVEF and total testosterone, and a negative association between the duration of hospital admissions and free testosterone. The relationships between BMI and testosterone levels have also been discerned in previous studies on healthy individuals. However, they have mainly reported an inverse relationship as obese men seem to have a lower serum testosterone level (29). This effect is attributed to the fact that higher testosterone induces more muscle mass and lower fat mass while DHEA-S also lowers lipid accumulation and triggers lipolysis (30). However, in the present study, an investigation of the association of BMI with testosterone levels in the participants with CAD revealed a positive correlation between BMI and free testosterone, but not total testosterone.

Furthermore, higher total testosterone levels were positively associated with LVEF, and higher free testosterone levels led to shorter hospitalization which confirms the beneficiary effect of testosterone on the participants' survival as suggested previously (16). Nevertheless, in our study, no death was reported during the admission to enable us to investigate the difference in the mortality or survival rate of the participants on different testosterone levels. Meanwhile, the duration of hospital stay or ICU admission failed to be associated with the testosterone levels.

This study investigated the difference in testosterone levels among the participants with CAD compared with a control group to indicate the effect of serum testosterone levels on CAD. However, this study had some limitations: firstly, the participants enrolment in this study were non-randomized, thus increasing the chance of bias in the results; secondly, we had no access to the information of the participants in the control group as we added this group after the completion of the study using laboratory results; and thirdly, we only measured the serum values of FSH, LH, and free and total testosterone but not of other hormones such as DHEA-S, estradiol, and sex-hormone-binding-globulin. Moreover, we did not have access to the angiographic results of the participants to investigate the difference in serum testosterone levels according to the severity of CAD in the participants.

## 5. Conclusion

The results of the present study demonstrated that participants with CAD bear a significantly low Testosterone level in comparison with the healthy control group as well as the association of the serum T levels with LVEF and negative relation with a hospital stay even though we observed no case of death in our study to investigate the exact effect of serum T levels on the participants' survival. Future studies with longer follow-up periods are required for the investigation of the effect of androgens on the survival of the participants with CAD and the difference in serum testosterone levels with regard to the severity of CAD.

##  Conflict of Interest

The authors declare no conflict of interest, financial or otherwise.

## References

[1] McAloon CJ, Boylan LM, Hamborg T, Stallard N, Osman F, Lim PB, et al. The changing face of cardiovascular disease 2000-2012: An analysis of the world health organisation global health estimates data. *Int J Cardiol* 2016; 224: 256–264. 10.1016/j.ijcard.2016.09.026 27664572

[2] NCD Countdown. 2030: Worldwide trends in non-communicable disease mortality and progress towards Sustainable Development Goal target 3.4. *Lancet* 2018; 392: 1072–1088. 10.1016/S0140-6736(18)31992-5 30264707

[3] Townsend N, Wilson L, Bhatnagar P, Wickramasinghe K, Rayner M, Nichols M. Cardiovascular disease in Europe: Epidemiological update 2016. *Eur Heart J* 2016; 37: 3232–3245. 10.1093/eurheartj/ehw334 PMC13480964 27523477

[4] Roth GA, Forouzanfar MH, Moran AE, Barber R, Nguyen G, Feigin VL, et al. Demographic and epidemiologic drivers of global cardiovascular mortality. *New Eng J Med* 2015; 372: 1333–1341. 10.1056/NEJMoa1406656 PMC4482354 25830423

[5] Piepoli MF, Hoes AW, Agewall S, Albus C, Brotons C, Catapano AL, et al. 2016 European guidelines on cardiovascular disease prevention in clinical practice: The sixth joint task force of the european society of cardiology and other societies on cardiovascular disease prevention in clinical practice (constituted by representatives of 10 societies and by invited experts) developed with the special contribution of the European Association for Cardiovascular Prevention & Rehabilitation (EACPR). *Atherosclerosis* 2016; 252: 207–274. 10.1016/j.atherosclerosis.2016.05.037 27664503

[6] Wong ND. Epidemiological studies of CHD and the evolution of preventive cardiology. *Nat Rev Cardiol *2014; 11: 276–289. 10.1038/nrcardio.2014.26 24663092

[7] Kiani F, Hesabi N, Arbabisarjou A. Assessment of risk factors in participants with myocardial infarction. *Glob J Health Sci* 2016; 8: 255–262. 10.5539/gjhs.v8n1p255 PMC4804079 26234995

[8] Pedersen LR, Frestad D, Michelsen MM, Mygind ND, Rasmusen H, Suhrs HE, et al. Risk factors for myocardial infarction in women and men: A review of the current literature. *Curr Pharmaceut Design* 2016; 22: 3835–3852. 10.2174/1381612822666160309115318 26956230

[9] Fairweather D. Sex differences in inflammation during atherosclerosis. *Clin Med Insights Cardiol* 2014; 8: S17068: 49–59. 10.4137/CMC.S17068 PMC4405090 25983559

[10] Perez-Lopez FR, Larrad-Mur L, Kallen A, Chedraui P, Taylor HS. Gender differences in cardiovascular disease: Hormonal and biochemical influences. *Reprod Sci* 2010; 17: 511–531. 10.1177/1933719110367829 PMC3107852 20460551

[11] Schwarz ER, Phan A, Willix Jr RD. Andropause and the development of cardiovascular disease presentation—more than an epi-phenomenon. *J Geriatr Cardiol* 2011; 8: 35–43. 10.3724/SP.J.1263.2011.00035 PMC3390065 22783283

[12] Xu L, Freeman G, Cowling BJ, Schooling CM. Testosterone therapy and cardiovascular events among men: A systematic review and meta-analysis of placebo-controlled randomized trials. *BMC Med *2013; 11: 108–120. 10.1186/1741-7015-11-108 PMC3648456 23597181

[13] Ahmadi A, Soori H, Sajjadi H, Nasri H, Mehrabi Y, Etemad K. Current status of the clinical epidemiology of myocardial infarction in men and women: A national cross-sectional study in iran. *Int J Prevent Med* 2015; 6: 14–18. 10.4103/2008-7802.151822 PMC4362287 25789146

[14] Hu X, Rui L, Zhu T, Xia H, Yang X, Wang X, et al. Low testosterone level in middle-aged male participants with coronary artery disease. *Eur J Int Med* 2011; 22: 133–136. 10.1016/j.ejim.2011.08.016 22075298

[15] Allameh F, Pourmand GH, Bozorgi A, Nekuie S, Namdari F. The association between androgenic hormone levels and the risk of developing coronary artery disease (CAD). *Iran J Public Health* 2016; 45: 14–19. PMC4822388 27057516

[16] Malkin CJ, Pugh PJ, Morris PD, Asif S, Jones TH, Channer KS. Low serum testosterone and increased mortality in men with coronary heart disease. *Heart* 2010; 96: 1821–1825. 10.1136/hrt.2010.195412 20959649

[17] Dellal FD, Mutlu Niyazoğlu T, Niyazoğlu M, Çeviker T, Görar S, Taşan E. Evaluation of androgen levels in participants with acute coronary syndrome. *Med Sci* 2012; 1: 323–330.

[18] Davoodi G, Amirezadegan A, Borumand MA, Dehkori MR, Kazemisaeid A, Yaminisharif A. The relationship between level of androgenic hormones and coronary artery disease in men. *Cardiovasc J Afr* 2007; 18: 362–366. PMC4170505 18092110

[19] Kloner RA, Carson C, Dobs A, Kopecky S, Mohler ER. Testosterone and cardiovascular disease. *J Am College Cardiol* 2016; 67: 545–557. 10.1016/j.jacc.2015.12.005 26846952

[20] Ruige JB, Mahmoud AM, De Bacquer D, Kaufman JM. Endogenous testosterone and cardiovascular disease in healthy men: A meta-analysis. *Heart* 2011; 97: 870–875. 10.1136/hrt.2010.210757 21177660

[21] Morris PD, Channer KS. Testosterone and cardiovascular disease in men. *Asian J Androl* 2012; 14: 428–435. 10.1038/aja.2012.21 PMC3720171 22522504

[22] Trost LW, Mulhall JP. Challenges in testosterone measurement, data interpretation, and methodological appraisal of interventional trials. *J Sex Med* 2016; 13: 1029–1046. 10.1016/j.jsxm.2016.04.068 PMC5516925 27209182

[23] Mattack N, Devi R, Kutum T, Patgiri D. The evaluation of serum levels of testosterone in type 2 diabetic men and its relation with lipid profile. *J Clin Diagn Res* 2015; 9: BC04–BC07. 10.7860/JCDR/2015/11049.5381 PMC4347063 25737972

[24] Moulana M, Lima R, Reckelhoff JF. Metabolic syndrome, androgens, and hypertension. *Curr Hypertens Reports* 2011; 13: 158–162. 10.1007/s11906-011-0184-0 PMC3820276 21274756

[25] Dhindsa S, Miller MG, McWhirter CL, Mager DE, Ghanim H, Chaudhuri A, et al. Testosterone concentrations in diabetic and nondiabetic obese men. *Diabetes Care* 2010; 33: 1186–1192. 10.2337/dc09-1649 PMC2875421 20200299

[26] Rao PM, Kelly DM, Jones TH. Testosterone and insulin resistance in the metabolic syndrome and T2DM in men. *Nat Rev Endocrinol* 2013; 9: 479–493. 10.1038/nrendo.2013.122 23797822

[27] Kelly DM, Jones TH. Testosterone: A metabolic hormone in health and disease. *J Endocrinol* 2013; 217: R25–R45. 10.1530/JOE-12-0455 23378050

[28] Kelly DM, Jones TH. Testosterone: A vascular hormone in health and disease. *J Endocrinol *2013; 217: R47–R71. 10.1530/JOE-12-0582 23549841

[29] Shamim MO, Ali Khan FM, Arshad R. Association between serum total testosterone and body mass index in middle aged healthy men. *Pak J Med Sci* 2015; 31: 355–359. 10.12669/pjms.312.6130 PMC4476341 26101490

[30] Gupta V, Bhasin S, Guo W, Singh R, Miki R, Chauhan P, et al. Effects of dihydrotestosterone on differentiation and proliferation of human mesenchymal stem cells and preadipocytes. *Mol Cell Endocrinol *2008; 296: 32–40. 10.1016/j.mce.2008.08.019 PMC2873614 18801408

